# The Role of Work as a Social Determinant of Health in Mother’s Own Milk Feeding Decisions for Preterm Infants: A State of the Science Review

**DOI:** 10.3390/children10030416

**Published:** 2023-02-21

**Authors:** Tricia J. Johnson, Paula P. Meier, Daniel T. Robinson, Sumihiro Suzuki, Suhagi Kadakia, Andrew N. Garman, Aloka L. Patel

**Affiliations:** 1 Department of Health Systems Management, Rush University, Chicago, IL 60612, USA; 2 College of Nursing, Rush University, Chicago, IL 60612, USA; 3 Department of Pediatrics, Rush University Medical Center, Chicago, IL 60612, USA; 4 Department of Pediatrics, Northwestern University Feinberg School of Medicine, Chicago, IL 60611, USA; 5 Department of Family and Preventive Medicine, Rush University Medical Center, Chicago, IL 60612, USA

**Keywords:** preterm infants, return to work, mother’s own milk, breastfeeding, breast milk, social determinants of health, mothers, health disparities, intergenerational transfer

## Abstract

In the United States, 10% of infants are born preterm (PT; <37 weeks gestational age) each year and are at higher risk of complications compared to full term infants. The burden of PT birth is borne disproportionately by Black versus non-Black families, with Black mothers significantly more likely to give birth to a PT infant. One proven strategy to improve short- and long-term health outcomes in PT infants is to feed mother’s own milk (MOM; breast milk from the mother). However, mothers must make decisions about work and MOM provision following PT birth, and more time spent in paid work may reduce time spent in unpaid activities, including MOM provision. Non-Black PT infants are substantially more likely than Black PT infants to receive MOM during the birth hospitalization, and this disparity is likely to be influenced by the complex decisions mothers of PT infants make about allocating their time between paid and unpaid work. Work is a social determinant of health that provides a source of income and health insurance coverage, and at the same time, has been shown to create disparities through poorer job quality, lower earnings, and more precarious employment in racial and ethnic minority populations. However, little is known about the relationship between work and disparities in MOM provision by mothers of PT infants. This State of the Science review synthesizes the literature on paid and unpaid work and MOM provision, including: (1) the complex decisions that mothers of PT infants make about returning to work, (2) racial and ethnic disparities in paid and unpaid workloads of mothers, and (3) the relationship between components of job quality and duration of MOM provision. Important gaps in the literature and opportunities for future research are summarized, including the generalizability of findings to other countries.

## 1. Introduction

In the United States, approximately 10 percent of infants are born preterm (PT; <37 weeks gestational age) [1], and prematurity is one of the leading causes of infant mortality [2]. The burden of prematurity is borne disproportionately by Black versus non-Black families, with a PT birth rate of 14.4% for Black mothers compared to 9.8% for Hispanic mothers and 9.1% for White mothers [1]. Furthermore, racial disparities persist after PT birth [3,4,5]. Although PT birth increases the risk for prematurity-related complications, including hospitalization, major neurodevelopmental disorders, and poor health compared to their term counterparts [6,7,8,9], Black PT infants are at an even greater risk for these prematurity-related complications compared to White PT infants [3,4,5]. While PT infants are a small proportion of total births, improving health outcomes and eliminating health disparities for PT infants is both a health and an economic priority due to the heightened risk for adverse health outcomes through childhood and into adulthood. 

### 1.1. Mother’s Own Milk Feedings as a Strategy to Improve PT Infant Health

PT infants, particularly those delivered at fewer than 32 weeks of gestation, are born with organs, including the brain, lung, and gut, that are not fully mature, and metabolic and immunomodulatory pathways that are underdeveloped [10,11]. These immature organs and underdeveloped pathways increase the risk for prematurity-related complications that often occur during the initial (i.e., birth) hospitalization, including late onset sepsis, with 7–14% incidence; necrotizing enterocolitis (NEC), with 4–7% incidence; and bronchopulmonary dysplasia, with 22–36% incidence [12]. Furthermore, these complications of prematurity increase the risk of adverse health and neurodevelopmental consequences in childhood and beyond and incur a cost to families and society overall through increased healthcare utilization and additional education needs [13,14,15,16,17,18,19,20,21,22,23]. One strategy to reduce the risk of these prematurity-related complications is feeding with mother’s own milk (MOM; breast milk from the mother, excluding donor human milk [DHM]), during the birth hospitalization [14,15,16,18,19,20,23,24]. A meta-analysis of observational studies that examined the dose-response relationship of human milk (i.e., MOM and/or DHM) in extremely PT (<28 weeks gestational age at birth) and very low birth weight (VLBW, <1500 g birth weight) infants, both higher-risk subgroups of all PT infants, reported that high-dose human milk was associated with a reduction in the risk of late-onset sepsis (relative risk [RR] 0.71, 95% confidence interval [CI] 0.56, 0.9), NEC (RR 0.53, 95% CI 0.42, 0.67), and bronchopulmonary dysplasia (RR 0.84, 95% CI 0.73, 0.96) [20,25]. Mechanistically, as described in a recent review, MOM programs the growth and development of body organs and pathways while simultaneously reducing the noxious impact of neonatal intensive care unit (NICU)-related stressors, including inflammation and oxidative stress [11]. These protective mechanisms are unique to MOM and are not shared by DHM nor commercial formula [24].

### 1.2. Disparities in the Receipt of Mother’s Own Milk Feedings by PT Infants

Large disparities exist in the receipt of MOM by PT infants, despite the demonstrated benefits. Although 78% of White and 82% Hispanic PT infants receive some MOM during the birth hospitalization, only 67% of Black PT infants receive any MOM during this critical time period [26]. Furthermore, research has shown that durations of MOM are also shorter for Black PT infants compared to non-Black PT infants, with only half as many Black infants receiving MOM at hospital discharge compared to non-Black infants [27,28,29,30]. Although exclusive MOM intake through 6 months of age is recommended by national and international health authorities, the lack of MOM at hospital discharge precludes achievement of this important milestone [20,31,32]. US national data on MOM intake by PT infants are not available, however, data for all infants demonstrate a large disparity at 6 months of age, with 45% of Black infants versus 62% of White infants receiving any MOM at this milestone [33]. Therefore, increasing MOM provision during the birth hospitalization can help in ultimately achieving exclusive MOM provision through 6 months of age. Despite national efforts to increase breastfeeding, the disparity between Black and White infants has continued to increase over the past decade, suggesting that these efforts are not addressing the underlying barriers to MOM provision [34]. Although a large body of research has examined the underlying causes of the Black-White disparity in PT birth, recently summarized in a review of the literature by Braveman et al. [35], less attention has been paid to understanding the underlying cause of disparities, and specifically the role of work as a social determinant of health, in MOM provision for PT infants. 

### 1.3. The Role of Work as a Social Determinant of Health

Social determinants of health are “the conditions in which people are born, grow, work, live and age” [36]. The role of paid work as a social determinant of health is well-documented, providing income and economic stability with implications for overall health and wellbeing [37]. In addition to providing a source of income, which may be the most recognizable way that work influences health, work impacts maternal health as a source of health insurance coverage and paid sick and parental leave [38,39]. However, other dimensions of work may also play an important role, such as job design, social conditions and social support, physical working conditions, and work schedule. For the vulnerable population of PT infants, maternal work and workload may contribute to health inequities, particularly as they relate to MOM provision. Although contemporary data are not available for mothers of PT infants specifically, one national survey found that 23% of women returned to work within 2 weeks and 44% returned to work within 8 weeks after childbirth [40]. US federal law guarantees up to 12 weeks off work through the Family Medical Leave Act (FMLA), however, this law guarantees time off work without pay, and only 11 states in the United States offer any paid family and medical leave [41]. Furthermore, FMLA does not apply to all workplaces with requirements for duration of work with the current employer (at least 12 months and 1250 h) and type of workplace (employed by a private company with ≥50 employees within 75 miles of the workplace, a public agency, or a private or public elementary or secondary school), thereby excluding half of private-sector and self-employed workers who are more likely to be female and Black or Hispanic [42,43]. In an analysis of paid leave using the 2017–2018 American Time Use Survey, only one-half of full-time workers had access to any paid leave for the birth of a child [44]. 

Although mothers with at least 12 weeks (3 months) of paid leave are significantly more likely to initiate MOM and provide MOM for longer durations than mothers without access to paid leave, only 50% of these mothers are still providing MOM at the recommended 6-month milestone, suggesting that returning to work may influence their MOM provision decisions [45]. In addition, while the Affordable Care Act of 2010 included a provision that requires accommodation of breastfeeding mothers in the workplace, studies evaluating the impact of this provision revealed a lower likelihood for low-income women and single mothers to have access to either break time or private space to breastfeed at work [46]. These studies also highlighted the potential for creating a two-tiered system of access to breastfeeding accommodations, due to employer discretion, that encourages employers to generously accommodate economically privileged mothers while leaving low wage mothers, especially African Americans, who have little bargaining power or resources unable to equally benefit from these accommodations [47]. As a structural determinant of health, work may support or hinder a woman’s decision and ability to provide MOM and it would be important to identify which aspects of work can be modified to facilitate the provision of MOM. A recent study that used national data on employed mothers for the first through fifth months after childbirth found that lower job control was associated with decreased breastfeeding up to six months postpartum and explained 31% of the Black-White difference in breastfeeding duration [48]. However, to our knowledge, none of these studies conducted to date specifically addressed the unique considerations faced by mothers of PT infants. 

### 1.4. Purpose of this Review 

Maternal decisions about work and MOM provision are made in the first few weeks and months after birth but have longer term implications for economic stability and infant health. However, there is a dearth of rigorous research on the relationship between work and MOM provision beyond paid parental leave and specific job characteristics, such as occupation. The purpose of this State of the Science review was to examine the research on the relationships between work, workload, and job quality with MOM provision for mothers of PT infants. Although a multitude of papers have examined the roles of paid time off and insurance coverage in maternal decisions about MOM provision [49,50,51,52], few studies have explicitly examined how other work-related factors and unpaid workload at home impact MOM decisions by mothers of PT infants. While paid parental leave is one factor that contributes to longer durations of MOM provision, mothers face additional barriers to MOM provision once they return to work, even for mothers who return to work more than 12 weeks after birth. The specific objectives were to (1) describe the complex decisions that mothers of PT infants make about returning to work, (2) synthesize the literature on racial and ethnic disparities in paid and unpaid workloads of mothers, (3) describe the relationship between components of job quality and duration of MOM provision, and (4) identify important gaps in the literature and opportunities for future research on the intersection of work, MOM provision, and health disparities, including the generalizability of findings to other countries. 

We searched PubMed and Scopus for peer-reviewed literature on racial and ethnic disparities, work, workload, and MOM provision by mothers of PT infants. For subtopics with few or no publications that reported findings for mothers of PT infants specifically, we expanded our search to include mothers of infants as a whole, including studies of term infants (gestational age ≥ 37 weeks), studies that included mothers of infants of all gestational ages and studies that included subsets of premature infants, such as very preterm (VPT, <32 0/7 weeks GA) infants and VLBW infants. Additionally, we hand-searched the references for additional peer-reviewed papers that we had not identified in our initial search. Due to a dearth of economic data for mothers of PT infants specifically, we reviewed US national data on overall maternal labor force participation, employment, and earnings by race and ethnicity. Although other factors such as antenatal steroids and coordinated evidence-based “Golden Hour” care administered in the first 60 min of life also impact health outcomes for PT infants [53,54], this review examines the role of MOM provision as a modifiable factor for improving PT infant health and how work as a social determinant of health may explain differences in MOM provision by maternal race and ethnicity [13,55,56,57].

## 2. The Interplay between Work and MOM Provision in Mothers of PT Infants 

### 2.1. PT Birth Creates Unique Circumstances for Mother and Infant That Impact Return to Work Decisions

The decisions made by mothers of PT infants about allocating time between paid and unpaid work (including whether to work at all after delivery) are more complicated than decisions for mothers of healthy term infants due to the unique circumstances of PT birth. PT infants have lengthy hospitalizations, ranging from less than 14 days on average for late preterm (LPT, 34 0/4–36 6/7 weeks gestational age [GA] at birth) to 20–30 days for moderate preterm (MPT, 32 0/7–33 6/7 weeks GA) to 65 days for VPT infants [18,58,59]. Although contemporary national statistics on the timing of return-to-work for mothers of PT infants are not available, in a 2012 survey of employees’ use of the FMLA, 23% of women returned to work within 2 weeks and 62% of women returned to work within 12 weeks after childbirth [40]. These data suggest that some mothers of PT infants may return to work before their PT infant is discharged from the hospital. Returning to work while providing MOM requires the use of a breast pump to continue lactation processes due to physical separation from the infant, and exclusive or partial breast pump use incurs significant out-of-pocket and time (opportunity) costs [60]. A very small proportion of women have health-related contraindications to providing MOM, thus considerations regarding returning to work and decisions about MOM provision affect most mothers of PT infants who plan to return to work [20,61]. Furthermore, mothers of PT infants disproportionately encounter other social risks that often coexist with higher rates of PT birth, such as poverty (as measured by enrollment in Medicaid and eligibility for the Special Supplemental Nutrition Program for Women, Infants, and Children), transportation barriers, and family structure (e.g., single head of household) that may impact MOM provision [11,27,28,29,30,62,63,64].

### 2.2. Unique Needs of PT Infants after Discharge from the Hospital That Impact the Unpaid Workload of Mothers

Once discharged from the hospital, PT infants may have frequent clinic and therapy visits and often need specialized care in the home [65,66]. For example, in a study of PT infants up to 24 months corrected age (i.e., age corrected for prematurity), 71% of infants had at least two clinic visits per month, 60% had at least one daily prescription medication, and 20% had durable medical equipment, such as oxygen, a feeding tube, or tracheostomy [66]. Additionally, three-quarters used early intervention services that require frequent therapy encounters, averaging between 1.8 to 4.4 h of therapy per month, depending on the type of service [66,67]. Apart from these health care needs, PT infants are susceptible to viral respiratory infections that may prevent attendance at a normal daycare facility, requiring families to utilize in-home childcare instead [68,69]. Although the gender gap in unpaid domestic work has narrowed over time, women continue to provide more childcare and household work and are more likely to take time off from work to care for sick children than men, even after accounting for paid workload [70,71]. Collectively, these findings suggest that mothers have additional responsibilities related to caring for their PT infant after being discharged from the hospital that compete with paid work and other unpaid household tasks. However, neither the burden of these responsibilities nor racial and ethnic differences in these responsibilities are well-documented. Furthermore, we found no data that have examined whether these responsibilities differ by race and ethnicity for mothers of PT infants.

### 2.3. Employment, Return to Work and MOM Provision after Childbirth 

The maternal decisions about work, including whether to return to work, and if so, when and for how many hours, have consequences for their income and economic stability, as well as their PT infant’s health. Although we did not find any research on the economic implications of MOM provision for mothers of PT infants specifically, research has demonstrated an inverse relationship between MOM provision and maternal earnings. In a national longitudinal study of employment and earnings by women who gave birth in the United States between 1980 and 1993, those who provided MOM for 6 months or longer had persistently lower earnings through 5 years after birth compared to mothers who either did not provide any MOM or provided MOM for less than 6 months [72]. The effect on earnings was attributed to mothers both reducing the number of hours worked and dropping out of the labor force altogether. These findings suggest there may be a trade-off between work and economic well-being with MOM provision, and racial/ethnic minority mothers, who are more likely to have low incomes, may not be able to afford the economic consequences of this trade-off [1,26,73].

Table 1 summarizes evidence on the relationship between work-related factors, including education and income due to their relationships with work, and MOM initiation and duration from the US perspective. Notably, full-time return to work (at least 35 h/week) has a large negative relationship with MOM provision [45,64,74,75,76]. Even after controlling for maternal education as another social determinant of health, research has demonstrated a negative relationship between return to work and MOM duration [45,76,77,78,79,80]. The vast majority of research, however, has either been limited to mothers of healthy, term infants or has subsumed mothers of PT infants into a larger sample of mothers without reporting separate findings. We located only one study that has explicitly examined return to work and MOM decisions of mothers with PT infants, although the study included a matched cohort of term infants in the analysis [64]. In their study of PT and term infants, Guendelman et al. found the odds of failing to initiate MOM provision were four times higher for mothers who return to work within 6 weeks after birth and more than two times higher for mothers who return to work between 6 and 12 weeks after birth compared to mothers who did not return to work by an average of 4.5 months after birth [64]. Additionally, the duration of MOM provision was significantly longer for mothers who did not return to work compared to those who returned to work. Taken together, research on maternal work decisions and MOM provision suggests these complex trade-offs have both economic and health implications, though studies have not explicitly examined racial and ethnic disparities in return to work and MOM provision for PT infants.

### 2.4. Unpaid Work and MOM Provision

Women bear a disproportionate share of unpaid work in the home (i.e., domestic services performed within a household that could be outsourced to someone outside of the household for pay, such as housework, yard work, running errands, and family care), and MOM provision is an additional mother-specific responsibility [84,85,86,87]. The “double burden” of work (i.e., paid work and unpaid work) that mothers who return to work experience [87] is further compounded by the additional needs of a PT infant [65,66], and mothers must decide how to allocate their scarce time between paid work, which provides income for the family, and unpaid work, including care of the PT infant and other family members. Although not specific to PT infants, one study of postpartum maternal workload reported that mothers (of primarily term infants) spend an average of 10.5 h per day providing care to the infant and other family members at 5 weeks postpartum, 7.2 h at 11 weeks, 4.7 h at 6 months, and 4.1 h at 12 months postpartum [86]. Although not separately reported, these figures include time spent providing MOM, which is considerable. One group of researchers found that breast pump-dependent mothers of infants born at <32 weeks gestational age or <1250 g birth weight spend 99 min per day providing MOM during the initial NICU hospitalization [88], and another group of researchers found that mothers who exclusively breastfed their (term) infant at 6 months of age spent 2.7 h in feeding-related activities per day [89]. Yet, these invisible contributions of unpaid work, in particular MOM provision, are rarely counted despite their recognized influence on overall health and well-being [87,90]. There are no data to our knowledge that explicitly compare unpaid workloads and MOM provision by maternal race and ethnicity, despite a potential link between racial and ethnic disparities in MOM provision and unpaid workload that we speculate may be tied to different poverty rates and marital status. Future research is needed to understand whether disparities in MOM provision for PT infants are explained by differences in unpaid workload.

### 2.5. Maternal Race/Ethnicity and Labor Force Experiences

Labor force data reveal large racial and ethnic gaps in employment and earnings that may ultimately contribute to decisions about providing MOM. Black mothers are more likely to be in the labor force (i.e., either working or not working but looking for work) than White mothers, and conditional on being in the labor force, are more likely to be unemployed rather than working [73]. In 2019, 78% of Black mothers were in the labor force, with 6.1% unemployed, compared to 72% of White mothers in the labor force, with 2.9% unemployed. Although fewer Hispanic mothers are in the labor force (65%), they are also more likely to be unemployed compared to White mothers. For mothers with children under age 3, the unemployment rates increase to 8.2% for Black and 5.2% for Hispanic mothers, compared to only 3.1% for White mothers [73]. Additionally, Black and Hispanic women continue to earn substantially less than White women, further magnifying economic disparities [73]. 

Compounding racial and ethnic disparities in employment and earnings, disparities also exist in paid time off (either paid sick leave or paid vacation leave), an essential benefit for new mothers that facilitates MOM provision for longer durations [91,92]. Overall, Black and Hispanic workers are more likely to be employed in jobs that do not offer paid time off compared to White workers, and the disparity is amplified by the occupational segregation of minority women into lower wage, lower quality jobs [93,94]. For example, only 51% of women employed in service occupations receive any paid time off, while 92% and 85% of women in managerial and professional occupations, respectively, are in jobs with paid time off. The differences in paid time off are even greater between high and low earners, with only 33% of women earning less than the federal poverty level having paid time off while 84% of women earning 400% or more of the federal poverty level have paid leave [93]. These differences in paid time off by occupation and earnings are a channel for exacerbating racial and ethnic disparities, given that larger proportions of Black and Hispanic women are employed in lower paying service occupations and smaller proportions are employed in higher paying managerial and professional occupations compared to White women [73]. 

### 2.6. Relationship between Job Characteristics and MOM Duration 

Although research on maternal work and MOM provision has focused predominantly on the availability of parental leave, other aspects of work may also play a role in supporting or undermining MOM provision [45,64,76,77,81,95]. This section briefly summarizes literature on the relationship between MOM provision and job characteristics which may relate to occupational segregation by race and ethnicity described in the prior section. Studies that have examined specific job characteristics and MOM provision, mainly in mothers of full-term, healthy newborns, demonstrate several important findings. (1) Higher work intensity (i.e., more hours worked) is associated with fewer breast milk feedings [75]. (2) Occupation plays a role in MOM provision, although inconsistencies in comparison groups make drawing conclusions about specific occupations difficult. For example, Dagher et al. found that mothers in professional jobs were significantly more likely to initiate MOM compared to mothers in clerical occupations, and Guendelman et al. found that mothers in managerial positions were more likely to initiate MOM compared to mothers in non-managerial positions [64,81]. (3) The evidence is mixed regarding the relationship between work status (working versus not working) and MOM provision, though household income may moderate the relationship. One study reported that mothers in service or labor occupations had significantly shorter durations of MOM provision compared to non-working mothers [78]. A separate study of low-income, predominantly minority mothers found that those in administrative and manual jobs had significantly shorter MOM durations when compared to non-working mothers but found no difference between mothers in either professional or service-related jobs and non-working mothers [80]. 

The mixed findings across studies may also be due to underlying heterogeneity in the types and nature of jobs classified within each occupational group by maternal race/ethnicity. A study by Whitley, Ro and Palma brings to light differences in the relationship between MOM duration and occupation by maternal race/ethnicity [78]. For White mothers, those who did not work had the longest duration of any amount of MOM provision (5.9 months), followed by working mothers in managerial or professional occupations (5.3 months) and working mothers in service or labor occupations (4.4 months). For Black mothers, however, those working in managerial or professional occupations had the longest durations (4.7 months) followed by working in service and labor occupations (3.1 months) and not working (3.0 months). (4) There is limited evidence that mothers with more perceived flexibility and control in their jobs have longer MOM durations [48,64]. A recent study of working conditions and duration of MOM provision found that low job control contributed to racial/ethnic disparities in the duration of MOM provision [48]. (5) Workplace support for MOM provision is a significant predictor of increased MOM duration [77,83]. These studies suggest specific job characteristics may support MOM provision while on-the-job; however, they may not adequately capture the multidimensional nature of the work environment and working conditions that may ultimately influence MOM provision, such as job quality. 

### 2.7. Job Quality as a Composite Measure of Work and Job Characteristics 

Over the past decade, two parallel strands of research have emerged that conceptualize and measure the multidimensional nature of work and its effect on worker health and may explain disparities in MOM provision by maternal race and ethnicity. The first strand focuses on job quality as a composite measure of working conditions, job characteristics, and work environment and comprises a variety of factors, such as work schedule and intensity, physical conditions, social conditions and social support, job security, and employment opportunities [96,97]. The second strand refers to “precarious employment,” and although there is not a single universally accepted definition, it generally refers to poor job quality [98,99,100,101,102]. These studies have shown cross-sectional relationships between job quality, precarious employment, and/or their components with overall worker health and wellbeing [96,102]. Steffgen, Sischka, and Fernandez de Henestrosa derived two composite measures of job quality using worker data from three European countries and found strong correlations between job quality and general well-being, presence of health problems, vigor (e.g., feeling energetic at work, looking forward to going to work), and work satisfaction [96], and in their scoping review, Gray et al. found that precarious employment was associated with poorer mental health [102]. Interestingly, neither Steffgen et al. nor any of the studies included in Gray et al’s scoping review took place in the United States. 

Research suggests that differences in job quality are not entirely attributed to occupation, and even within occupations, racial and ethnic disparities in job quality remain. For example, Black workers in service occupations are more likely to be employed in low quality jobs compared to White workers in similar occupations [94], suggesting segregation into low quality jobs within broader occupational classes. Furthermore, the occupational segregation of minority mothers into certain occupations, such as service-related jobs, may lead to employment in lower quality jobs that undermine MOM provision. A recent study of employment and MOM provision using data for women who gave birth between 2007 and 2015 from the Panel Study of Income Dynamics (PSID) found that job control, a component of job quality, mediates the relationship between maternal race/ethnicity and duration of MOM provision [48]. However, this study did not report information for mothers of PT infants specifically. Additionally, the PSID asked mothers about their duration of any MOM provision up to five years after childbirth, limiting their analysis to any MOM (rather than exclusive MOM) at 6 months of age, thereby combining exclusive MOM with mixed MOM-formula feedings. Therefore, there is a need to more precisely quantify the duration of exclusive MOM provision, which requires more time during the workday, by mothers of PT infants to understand whether job quality mediates observed disparities in MOM provision among Black and non-Black mothers of PT infants.

## 3. The Gaps in Science

Although exclusive MOM intake through six months of age is the gold standard, a large Black-White disparity in MOM feedings of PT infants exists. As described by the World Health Organization, the conditions in which people work are an important social determinant of health that shape everyday life [36] and may explain racial disparities in MOM provision. However, rigorous research is needed to address key gaps in the science related to work and racial disparities of MOM provision for PT infants.

### 3.1. Little Information Is Available about the Role of Work in Decisions about MOM Provision for PT Infants 

Little information is available about the role of work in creating disparities for mothers and their PT infants. To our knowledge, only one cohort of mothers of PT infants has been examined with respect to return to work and MOM decisions, and the studies reflect both births and maternal employment decisions two decades ago (2002–2003) and the analytic sample combined working mothers of PT and term infants, rather than analyzing them separately [64,103]. Mothers of PT infants who are entirely or partially breast pump-dependent need to pump frequently to maintain an adequate MOM supply, and inadequate break times and low-quality breast pumps are barriers to maintaining an adequate MOM supply for these mothers [104]. Combined employment stress and concerns about infant health downregulate an effective prolactin response, potentiating MOM volume problems [105,106]. Previous research has neither comprehensively quantified these needs in a single study nor examined whether high quality jobs can help mothers address barriers to continued MOM provision once back on the job. Given the racial disparities in MOM feedings of PT infants and the documented racial differences in work conditions and benefits, future research should examine whether disparities in MOM feedings can be explained by racial differences in job quality and the timing of return to work by mothers of PT infants, or other unexamined work factors. In addition, a national study quantifying the percentage of mothers of PT infants who are working, stratified by race and ethnicity, is also warranted.

### 3.2. Unpaid Work Is Largely Ignored as a Determinant of MOM Provision

To date, the science of work and MOM provision has narrowly defined work as “paid work,” thereby ignoring both the burden of unpaid work and mothers who are not in the labor force. The accurate measurement of the unpaid time spent in MOM provision will provide policymakers, health insurers, and healthcare providers more rigorous information about the investments required to improve MOM feedings in this vulnerable population. Little attention has been paid to potential racial and ethnic inequities in the burden of unpaid work for mothers. It is likely that the consequences of the inequitable burden of unpaid work manifests as disparities in MOM provision. Whitley, Ro, and Palma found nonworking White mothers had the longest durations of MOM provision, while nonworking Black mothers had the shortest durations of MOM provision [78]. This dichotomy points toward unpaid workload as a promising line of inquiry for understanding disparities in MOM provision. Although not measured, it is possible that not working may be due to different economic circumstances for White and Black mothers resulting in differing unpaid workload responsibilities. Additionally, while the concept of total workload (paid + unpaid work) is not new, the role of total workload in explaining racial disparities in the receipt of MOM in PT infants has not been explored [86,107,108,109].

### 3.3. The Trade-Offs That Mothers of PT Infants Make about Paid Work, Unpaid Work and MOM Provision Are Not Well Understood 

In addition to quantitative studies that examine the relationships between work and MOM provision, there is a research gap in understanding how mothers make decisions and how they weigh competing demands for their time. Research in the NICU has demonstrated that disparities in MOM provision occur over the course of the birth hospitalization because Black and non-Black mothers initiate MOM provision at similar rates, with Black mothers especially likely to change the feeding decision from formula to MOM with provider education [110]. However, regardless of geographical location, rates of MOM provision at NICU discharge are significantly lower for Black than non-Black mothers [28,111]. These results suggest that disparities in MOM provision are not due to a lack of maternal understanding about the importance of MOM, but instead are due other factors, such as the opportunity cost of providing MOM, which differ for Black mothers who may encounter more economic and workplace-related barriers to providing MOM than White mothers. While the benefits of MOM are similar across infants, after controlling for infant gestational age and severity of illness, the opportunity cost of providing MOM may be higher for women who are low income and with higher paid and unpaid workloads. Understanding these decision-making processes among mothers across different socioeconomic measures and race/ethnicity, particularly in light of the need to weigh immediate costs and benefits with longer-term, uncertain trade-offs, is a priority. For example, not returning to work has an immediate and certain negative impact on income, while not providing MOM has a potential, but uncertain deleterious impact on infant health in the future, and both decisions will influence the risk of the intergenerational transfer of disparities. Differences in the opportunity costs and time horizons or “delay discounting” (i.e., subjective weighting of immediate versus future benefits) may explain disparities in MOM provision, but this area of research is uncharted territory.

### 3.4. A Global Perspective on the Interplay of Work and Workload on MOM Provision Decisions Is Needed

Although this review primarily focused on disparities in MOM provision and the roles of work and workload from a US perspective, many of the issues discussed in this review have global relevance. Research has shown that maternal employment is associated with shorter durations of MOM provision in upper middle income countries, such as China and Mexico [112,113,114,115]. Even in high income countries with mandatory paid maternity benefits, such as Australia and South Korea, mothers who return to work have shorter MOM durations compared to mothers who do not work after birth [79,116]. Additionally, similar to the evidence from the United States, shorter intervals between birth and return to work are also associated with shorter MOM durations in countries including France, Scotland, and Hong Kong [117,118,119,120]. Although we found no studies outside of the US that explicitly examined unpaid workload and duration of MOM provision, working women in European countries spend three to four hours per day in unpaid care work within the household, suggesting that unpaid work and total workload are likely to be barriers to MOM provision in these countries as well. Although European and other Organisation for Economic Co-operation and Development countries guarantee some paid maternity leave, paid leave ends prior to the critical 6 months of life milestone for the vast majority of these countries [121], and therefore, competing decisions about paid and unpaid work versus MOM provision may also be important to consider [122]. Evidence from across the world points to work as a potentially important social determinant of health that may act as a barrier to the long duration of MOM provision; however, policymakers and healthcare providers need more rigorous research that pinpoints the specific aspects of work that may hinder or facilitate longer MOM provision in order to design effective mechanisms.

## 4. Conclusions

Although many of the findings related to work, workload, and MOM provision in this review apply to all infants, PT infants are more susceptible than healthy term infants to neonatal complications of prematurity, respiratory problems, poor neurodevelopment, and rehospitalization. These adverse outcomes are mitigated with MOM; however, disparities in MOM provision exist that may worsen health disparities through childhood. Federal agencies, including the National Institutes of Health in the United States, have recognized the role of work as a social determinant of health and the need for more research to understand the underlying mechanisms by which work creates or intensifies disparities [123]. Rigorous observational and mechanistic studies are needed to evaluate the specific components of paid and unpaid work that contribute to disparities in MOM provision in the vulnerable population of PT infants. The occupational segregation of minority women into lower quality and lower paid jobs may not only play a role in economic disparities for mothers themselves, but also create health and economic disparities for their PT infants. An abundance of research has shown that intervening early in life to improve health and eliminate disparities has lifelong consequences, and with the exception of prenatal care, few interventions occur earlier in life than MOM provision.

## Figures and Tables

**Table 1:** Summary of work-related factors associated with MOM initiation and duration.

Variable	MOM Initiation	MOM Duration
Occupation	- Mothers in professional occupations were more likely to initiate MOM compared to mothers in clerical occupations [81]	- Mothers in administrative and manual occupations had higher odds of MOM cessation compared to mothers who stayed at home [80] - Relative risk of MOM cessation was lower for mothers in managerial occupations compared to mothers in other occupations [64] - Relative risk of MOM cessation was higher for mothers in professional occupations compared to mothers in clerical occupations [81] - Mothers working as farmers, artisans, and merchants were more likely to continue MOM provision after returning to work compared to mothers working as managers; mothers classified as intermediate employees and manual workers were less likely to continue MOM provision after returning to work compared to mothers working as managers [82] - Mothers in service/labor occupations had shorter MOM durations compared to mothers who were not working [78]
Maternity leave duration	- Mothers who had not yet returned to work had greater odds of MOM initiation compared to mothers who returned to work in 1 to 6 weeks after birth [76]	- Mothers who returned to work in the prior month, current month, or next month had greater odds of MOM cessation compared to mothers not working in the month [80] - The relative risk for MOM cessation was higher for mothers who returned to work compared to mothers who had not returned to work [64] - Returning to work more than 12 weeks after birth was associated with greater odds of predominant MOM for more than 3 months compared to returning to work in 1–6 weeks [76] - Duration of work leave was positively associated with duration of any MOM and duration of exclusive MOM [75] - Relative risk of MOM cessation was higher for working mothers compared to mothers on leave from work [81] - Time off work was positively associated with the probability of near-exclusive MOM trajectory through 12 weeks after birth [77]
Work hours		- Mothers who returned to work within 6 months at full-time employment or returned to work between 3 and 6 months with part-time or casual employment had lower odds of breastfeeding at 6 months compared to mothers who were not employed at 6 months [79] - Mothers who worked part time or full time and returned to work within 12 weeks had a shorter MOM duration, compared to mothers who did not work in the 12 weeks after birth [74] - Returning to work full time was associated with higher odds of not providing MOM for at least 3 months compared to not working at 3 months [45] - Compared to not working at 3 months after childbirth, returning to work full time before 3 months postpartum was associated with higher odds of not meeting the mother’s intention of providing MOM for at least 3 months [45]
Income	- Income was positively associated with odds of MOM initiation [80]	- Household income was negatively associated with the probability of near-exclusive MOM trajectory through 12 weeks after birth [77]
Maternal education	- Having a high school education or more education was associated with higher odds of MOM initiation compared to having less than a high school education [80] - Having a bachelor’s degree or more education was associated with higher odds of MOM initiation compared to having less than a bachelor’s degree [78]	- Having a high school degree or more education was associated with lower odds of MOM cessation compared to not having a high school degree [80] - Having a secondary education or more education was associated with higher odds of breastfeeding at 6 months after birth compared to not completing secondary education [79] - Having a high school degree or less was associated with shorter MOM duration compared to having a college degree or more education [74] - Greater maternal education was associated with higher probability of near-exclusive MOM trajectory through 12 weeks after childbirth [77] - Having a trade school education or higher education less than a bachelor’s degree was associated with higher odds of MOM at NICU discharge compared to having less than a high school degree [27] - Having less than a graduate degree was associated with shorter MOM duration compared to having a graduate degree [83]

## Data Availability

Not applicable.
